# Protein quantification and visualization *via* ultraviolet-dependent labeling with 2,2,2-trichloroethanol

**DOI:** 10.1038/s41598-019-50385-9

**Published:** 2019-09-26

**Authors:** Anand Chopra, William G. Willmore, Kyle K. Biggar

**Affiliations:** 1 Department of Biology, Carleton University, 1125 Colonel By Dr, Ottawa, ON K1S 5B6 Canada; 2 Institute of Biochemistry, Carleton University, 1125 Colonel By Dr, Ottawa, ON K1S 5B6 Canada; 3 Department of Chemistry, Carleton University, 1125 Colonel By Dr, Ottawa, ON K1S 5B6 Canada

**Keywords:** Proteins, Biochemical assays, Proteomic analysis

## Abstract

The incorporation of 2,2,2-trichloroethanol in polyacrylamide gels allows for fluorescent visualization of proteins following electrophoresis. Ultraviolet-light exposure, in the presence of this trichlorinated compound, results in a covalent modification of the tryptophan indole ring that shifts the fluorescent emission into the visible range. Based on this principle, we used 2,2,2-trichloroethanol to develop a microplate format protein quantification assay based on the fluorescent signal generated by modified proteins. We also demonstrated a specific fluorescent emission of 2,2,2-trichloroethanol-labeled protein at 450 nm, with a 310 nm excitation, resulting from modification of both tryptophan and tyrosine residues. Following optimization, this protein quantification assay displayed superior sensitivity when compared to UV absorbance at 280 nm (A280), and enabled quantification beyond the linear range permitted by the Bradford method. This 100 μL assay displayed a sensitivity of 10.5 μg in a range up to at least 200 μg. Furthermore, we extended the utility of this method through the development of a 20 μL low-volume assay, with a sensitivity of 8.7 μg tested up to 100 μg, which enabled visualization of proteins following SDS-PAGE. Collectively, these results demonstrate the utility of 2,2,2-trichloroethanol-based protein quantification and demonstrates the protein visualization in polyacrylamide gels based on 2,2,2-trichloroethanol-labeling pre-electrophoresis.

## Introduction

The purification of protein is an essential step in many molecular biology and biochemistry workflows and laboratory practices often require a rapid and sensitive method for subsequent quantification. Most commonly used protein quantification methods include, but are not limited to, the standard Bradford^1^ and Lowry^2^ assays, as well as UV absorbance measurements at 280 nm (i.e., A280). Collectively, these assays all exploit the physiochemical properties of proteins to generate a detectable and quantifiable signal that is proportional to protein concentration. Additionally, these assays are rapid and provide convenience such that they can be typically performed in a variety of formats.

**Figure 1:**
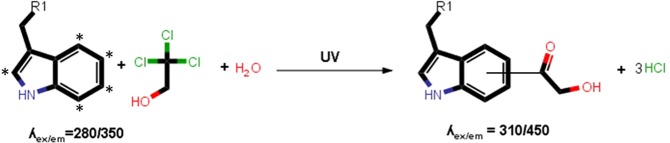
Reaction of TCE with tryptophan residues yields multiple monosubstituted isomers of an acylated indole ring. R1 denotes the peptide backbone and stars indicate possible sites of attachment The reaction was inferred from literature^5,6^.

It has now been well-established that trichlorinated compounds are able to react with proteins upon exposure to UV-light, generating modified tryptophan residues with red-shifted fluorescent properties^3,4^. This chemistry has been successfully applied within polyacrylamide gels to fluorescently label protein post-electrophoresis by adding low levels of 2,2,2-trichloroethanol (TCE) to the gel matrix^3,4^. Chloroform and trichloroacetic acid (TCA) were the first trichlorinated compounds used in this manner and were incorporated into polyacrylamide gels following electrophoresis through soaking of the gel in solutions containing either of these individual compounds^3^. Subsequently, the immediate visualization of proteins post-electrophoresis was enabled through incorporation of TCE as a component of polyacrylamide gels prior to gel casting^4^. Following a timed exposure to UV, each of these reactions result in the covalent modification of tryptophan residues. Specifically, chemical groups are attached to the indole ring, and in the case of TCE, the photomodification is acylation of the ring^5,6^ (Fig. 1). It should be noted the reactions with halocompounds result in the formation of multiple monosubstituted isomers as attachment of the chemical group may occur on the 2, 4, 5, and 6 carbons of the indole ring, as demonstrated by the reaction of chloroform with tryptophan residues^5^.

The UV-induced modification of tryptophan residues with trichlorinated compounds has allowed for development of “stain-free” technologies used for the visualization of proteins on a UV-transilluminator immediately after electrophoresis. Protein fluorescence can then be monitored as a result of a red-shifted emission spectrum that extends into the visible range. Upon photomodification of tryptophan residues with trichlorinated compounds, the natural indole tryptophan peak fluorescence emission at 350–360 nm diminishes^3,7^. However, tryptophan residues modified with TCA, TCE, or chloroform have demonstrated increased peak fluorescence emission at wavelengths above 400 nm^3,7^. Applications for chemistry also include protein quantification within polyacrylamide gels and total protein normalization for western blot analysis^8–11^. Furthermore, the TCE-tryptophan reaction has been applied towards fluorescence enhancement for the detection of protein crystals and the determination of tryptophan residue solvent accessibility^12,13^.

Additional applications of TCE-modified proteins have been limited to date, however, the use of this chemistry for protein detection and quantification is of broad research appeal. In this study, the fluorescent properties of acylated tryptophan residues, produced by the photochemical modification of tryptophan with TCE, were exploited to develop a microplate format protein quantification assay. We demonstrated that TCE undergoes reactions with both tryptophan and tyrosine amino acids to yield products with enhanced fluorescence emission in the 350–600 nm range. Furthermore, the TCE assay displayed advantages over traditional protein quantification assays; the Bradford method and A280 assay. Finally, we modified our assay to work in a low-volume format, enabling practical re-use of the protein sample for SDS-PAGE analysis.

## Materials and Methods

### Reagents

Glycine (Sigma; G7403), L-phenylalanine (Fischer Scientific; BP391), L-tryptophan (Fischer Scientific; BP395), L-tyrosine (Fisher Scientific; BP396), 2,2,2-trichloroethanol (Sigma-Aldrich; T54801), Bovine Serum Albumin (BSA; BioShop; ALB001).

### Solution preparation

BSA standards between 0–20 μg/μL were prepared in phosphate-buffered saline (PBS) (137 mM NaCl, 2.7 mM KCl, 10 mM Na_2_HPO_4_, 1.8 mM KH_2_PO_4_) from the highest concentrated standard (20 μg/μL). Similarly, glycine, L-phenylalanine, and L-tryptophan solutions of 0–1 μg/μL and L-tyrosine solutions of 0–0.4 μg/μL were prepared in PBS.

### Protein quantification

The Bradford method was utilized with few modifications indicated^1^. To 2 μL of protein sample, 198 μL of Bradford reagent (0.1 mg/mL Coomassie Brilliant Blue G-250, 5% (v/v) methanol, 8.5% H_3_PO_4_) was added and incubated at room temperature for 5 minutes. Absorbance was measured at 595 nm. For UV A280 experiments, the absorbance at 280 nm of 100 μL BSA standard solutions was measured in a UV-transparent microplate.

All experiments involving the reaction of TCE with proteins or amino acids followed the same workflow: samples were first incubated with TCE under a 15 W UV-lamp followed by fluorescent measurements with a BioTek Cytation 5 microplate reader. The initial characterization of the fluorescence emission of the reaction products (emission ʎ = 350–600 nm and excitation ʎ = 310 nm) and excitation spectra (emission ʎ = 450 nm and excitation ʎ = 250–400 nm) utilized 5-minute incubations of the samples with a final assay concentration of 0.5% (v/v) TCE under UV-light. To achieve this, 90 μL of TCE reagent (0.56% (v/v) TCE in PBS) was incubated with 10 μL of 0–1 μg/μL BSA, glycine, L- phenylalanine, and L-tryptophan as well as 0–0.4 μg/μL L-tyrosine.

Optimization of TCE concentration and UV-exposure time were done using BSA protein as a standard at final assay concentrations of 0.5 µg/µL and 1.0 µg/µL. TCE concentration optimization was performed with a 5-minute UV-exposure time and final assay concentrations of 0–2% (v/v) TCE, followed by fluorescence intensity readings (emission ʎ = 450 nm and excitation ʎ = 310 nm; emission ʎ = 280 nm and excitation ʎ = 350 nm). Optimization of UV-exposure time with the 15 W UV-lamp utilized a final assay concentration of 0.5% (v/v) TCE and 0–30-minute incubation while monitoring the fluorescence emission at ʎ = 450 nm with an excitation at ʎ = 310 nm.

The final TCE assay incorporated the same volumes of TCE reagent and sample described previously, resulting in a final TCE assay concentration of 0.5%, and a 15-minute UV-exposure time followed by the measurement of fluorescence emission at ʎ = 450 nm with an excitation at ʎ = 310 nm.

### Low-volume TCE assay and SDS-PAGE visualization

The total volume of the TCE assay was reduced to 20 μL while maintaining the same volumes of sample and original TCE stock solution. To 10 μL of protein solution, an equal volume of TCE Ultra Reagent (5% (v/v) TCE in PBS) was added followed by 0–15 minutes of UV-exposure while monitoring the fluorescence emission at ʎ = 450 nm with an excitation at ʎ = 310 nm. Subsequently, the assay was diluted in 2 X Laemmli Sample Buffer (Bio-Rad Laboratories), heated at 95 °C for 5 minutes, and 30 μL of diluted solution (i.e. 75% of total protein) was subjected to SDS-PAGE on a 10% resolving gel at 120 V for 1.5 hours. The fluorescence of TCE-modified protein was visualized directly on a BioRad ChemiDoc^TM^ XR+ System using a UV-light box. Furthermore, proteins in the same gel were visualized after incubations of the gel in Coomassie Brilliant Blue (CBB) staining solution (1 mg/mL CBB R250, 50% (v/v) methanol, 10% (v/v) glacial acetic acid) and destaining solution (50% (v/v) methanol, 10% (v/v) glacial acetic acid).

## Results and Discussion

### Protein fluorescent spectra

The majority of protein quantification methods exploit chemical interactions between reagent compounds and proteins, generating detectable and quantifiable signals that are proportional to concentration. The Bradford method and Lowry assay are two widely used protein quantification assays that result in colorimetric shifts, enabling quantification based on absorbance^1,2^. These assays are based on the colour changes of bound vs unbound anionic dyes (i.e. CBB) and copper-based reactions, respectively. The TCE-based methodology presented herein extends the range of rapid and sensitive protein quantification methods that are currently available to researchers.

Detection and quantification of proteins in polyacrylamide gels with trichlorinated compounds relies on UV-dependent covalent modification of the tryptophan indole ring with chemical groups derived from such trichlorinated compounds^3,4^. This modification results in a red-shifted fluorescent emission extended into the visible range, allowing for visualization of protein bands with a gel imaging system. Specifically, the reaction involving TCE yields an acylated indole ring (Fig. 1) and has been used widely in visualization of protein in polyacrylamide gels^3,4^. The TCE assay described herein exploits this photochemical modification to generate a quantification curve for protein concentration as a function of TCE-reacted protein fluorescence. BSA was chosen as the analytical standard as it is commonly used for this purpose across different quantification methods and also has a typical ~3% tryptophan residue content.

**Figure 2:**
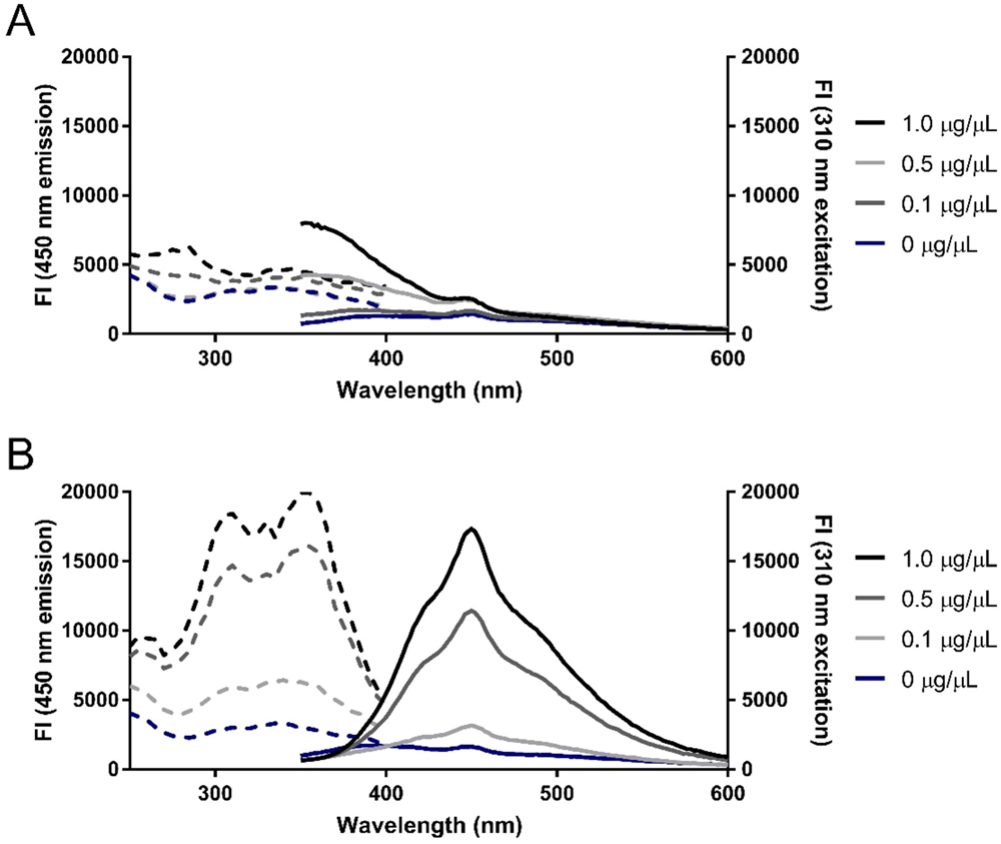
Excitation (dashed) and emission (solid) spectra of unreacted and TCE-reacted BSA. Fluorescence intensity (FI) of 0–1 µg/µL BSA following 5 minutes of UV-exposure in the (**A**) absence or (**B**) presence of 0.5% (v/v) TCE. Emission spectra (emission ʎ = 350–600 nm; excitation ʎ = 310 nm) and excitation spectra (emission ʎ = 450 nm; excitation ʎ = 250–400 nm) were monitored in a BioTek Cytation 5 microplate reader.

Initially, the fluorescent properties of the reaction products were characterized prior to optimization of TCE percentage and UV-exposure time. For this purpose, 0.5% (v/v) TCE was used as this percentage was demonstrated to be optimal in SDS-PAGE band detection^4^. Additionally, from lab usage and previous literature^4^ it is known that signals in SDS-PAGE generally appear and saturate within less than 5 minutes of UV-exposure, therefore a 5-minute reaction time was used for all subsequent experiments involving characterization of protein fluorescence. At an excitation of 310 nm, the emission monitored between 350–600 nm displayed a maximum at 450 nm, which was absent in unreacted protein (Figs 2 and S1). Furthermore, the fluorescence intensity at 450 nm increased in a dose-dependent manner with protein concentration (Fig. 2B). Natural tryptophan fluorescence at an emission of 350 nm could be observed in unreacted protein, whereas the fluorescence at this wavelength greatly diminished in the presence of 0.5% (v/v) TCE (Fig. 2). Although TCE has been demonstrated to be a quencher of protein fluorescence in the absence of intentional UV exposure^14^, the shift of peak fluorescence emission from 350 nm to 450 nm upon incubation with TCE under UV-exposure is an indicator of tryptophan labeling.

The incubation of BSA with TCE altered the excitation spectra monitored between 250–400 nm at a 450 nm fluorescence emission (Fig. 2). Within these parameters, TCE-reacted BSA displayed increasing fluorescence intensity with protein concentration, whereas unreacted BSA failed to be reliably detected. More specifically, the former displayed two observable peaks at 310 nm and 355 nm excitation wavelengths with comparable fluorescence intensities at 450 nm emission (Fig. 2B). Similarly, excitation peaks at 315 nm and 355 nm for TCE-reacted calf-unwinding protein (UP1) had previously been observed at a 455 nm emission^7^. Although one excitation peak was anticipated due to the number of applications in literature referring to the use of TCE as a label for only the tryptophan indole ring, we speculated that other UV-excitable residues may react with TCE to yield additional products with red-shifted fluorescent properties. Indeed, *N*-acetyltyrosineamide has been demonstrated to react with TCE and yield a product with altered fluorescent spectra^14^. Therefore, the two excitation peaks result from reactions of TCE with both tryptophan and tyrosine residues to yield semi-overlapping fluorescent spectra which vary between proteins due to variance in amino acid content. This may be confirmed by obtaining the fluorescent spectra of TCE-reacted proteins that lack either tryptophan or tyrosine residues.

### Amino acid reactivities

**Figure 3:**
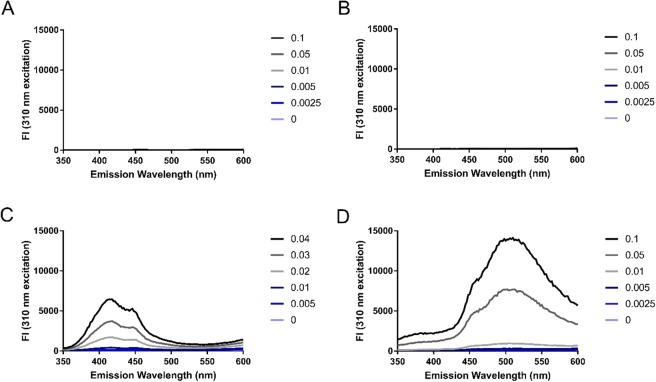
Emission spectra of individual amino acids following UV-exposure in the presence of TCE. Emission spectra (emission ʎ = 400–600 nm; excitation ʎ = 310 nm) of (**A**) glycine, (**B**) phenylalanine, (**C**) tyrosine, and (**D**) tryptophan amino acids were collected following 5 minutes of UV-exposure in the presence of 0.5% (v/v) TCE. Figure legends indicate the concentration of amino acids in µg/µL.

The reaction of TCE with tryptophan is dependent on the indole ring entering an excited electron state, which is attainable via UV-irradiation. Other aromatic amino acids such as phenylalanine and tyrosine are also UV-excitable, therefore we explored the reactivity of TCE with aromatic amino acids through the generation of products with fluorescent emission between 350–600 nm. As two observable peaks were present in the excitation spectra of TCE-reacted protein at 310 nm and 355 nm (Fig. 2B), we examined the TCE-reacted amino acid emission spectra at both excitation wavelengths. At 310 nm excitation, the fluorescence emission intensity of TCE-reacted tyrosine and tryptophan increased in a dose-dependent manner with amino acid concentration (Fig. 3C,D), whereas incubation of TCE with glycine and phenylalanine under UV-light did not yield any detectable fluorescent products at measured concentration (Fig. 3A,B). Additionally, only TCE-reacted tryptophan displayed an observable dose-dependent relationship between amino acid concentration and fluorescence emission intensity at 355 nm excitation (Fig. S2). In agreement with the fluorescent emission spectra of unreacted and TCE-reacted BSA, unreacted tyrosine and tryptophan amino acids displayed little to no observable fluorescence in comparison to the corresponding TCE-reacted amino acids at peak wavelengths (Fig. S3). As 310 nm excitation light yields fluorescent signal from both TCE-reacted tryptophan and tyrosine residues, this excitation wavelength was used in subsequent experiments as it allows for quantification based on multiple amino acid residues.

### Assay optimization

**Figure 4:**
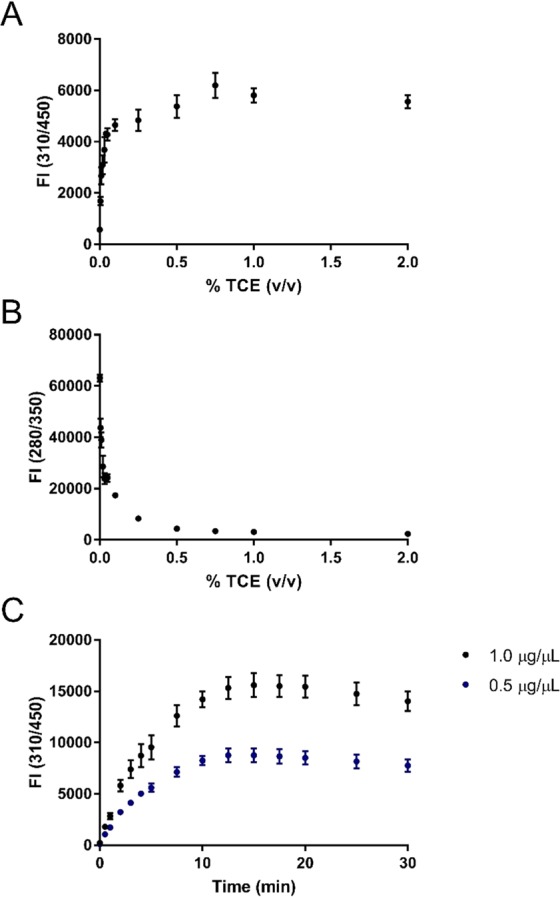
TCE concentration and UV-exposure time optimization. Fluorescence intensity (FI) of (**A**) modified (emission ʎ = 450 nm; excitation ʎ = 310 nm), and (**B**) unmodified protein (emission ʎ = 350 nm; excitation ʎ = 280 nm) in 0.5 µg/µL BSA following 5 minutes of UV-exposure in the presence of 0–2% (v/v) TCE. (**C**) Effect of UV-exposure time on FI measurements (emission ʎ = 450 nm; excitation ʎ = 310 nm) of 0.5 µg/µL and 1.0 µg/µL BSA following 0–30 minutes of UV-exposure in the presence of 0.5% (v/v) TCE. Error bars represent propagated standard deviations of blank corrected means.

Following characterization of UV-dependent TCE-labeling of protein and amino acids, a re-optimization of TCE concentration and UV-exposure time was performed to determine optimal reaction conditions providing maximal sensitivity at 310/450 nm emission/excitation fluorescence (i.e. TCE-reacted protein fluorescence). Initially, TCE concentration was varied between 0–2% (v/v) while fixing UV-exposure time at 5 minutes and protein concentration at 0.5 µg/µL. TCE-reacted protein fluorescence increased in a dose-dependent manner with TCE concentration to a maximal signal and plateau after 0.1% (v/v) TCE (Fig. 4A). An opposing relationship was observed for natural indole fluorescence, plateauing to a minimum after 0.5% (v/v) TCE (Fig. 4B). The decrease in natural indole fluorescence was expected as covalent modification of the tryptophan ring with trichlorinated compounds results in a shifted fluorescent emission, as was demonstrated through comparisons of unreacted and TCE-reacted emission spectra (Fig. 2). Thus, a final TCE concentration of 0.5% (v/v) was chosen for subsequent experiments as minimal natural indole fluorescence and maximal TCE-reacted protein fluorescence are ideal criteria to be satisfied. Finally, UV-exposure time was varied between 0–30 minutes, while fixing TCE concentration constant at 0.5% (v/v) and protein concentration at 0.5 µg/µL and 1.0 µg/µL. An optimal UV-exposure time of 15 minutes was chosen for subsequent experiments as maximal TCE-reacted protein fluorescence occurred at this time point (Fig. 4C). Furthermore, it could be observed that 0.5% (v/v) TCE was in excess for both 0.5 µg/µL and 1.0 µg/µL protein as the TCE-reacted protein fluorescence plateaued in the same time frame for both protein concentrations. Although further optimization for TCE concentration using a 15-minute UV-exposure time would likely demonstrate an optimal concentration lower than 0.5% (v/v), using this concentration ensures the reagent is sufficiently available in excess.

### Quantification curves

**Figure 5:**
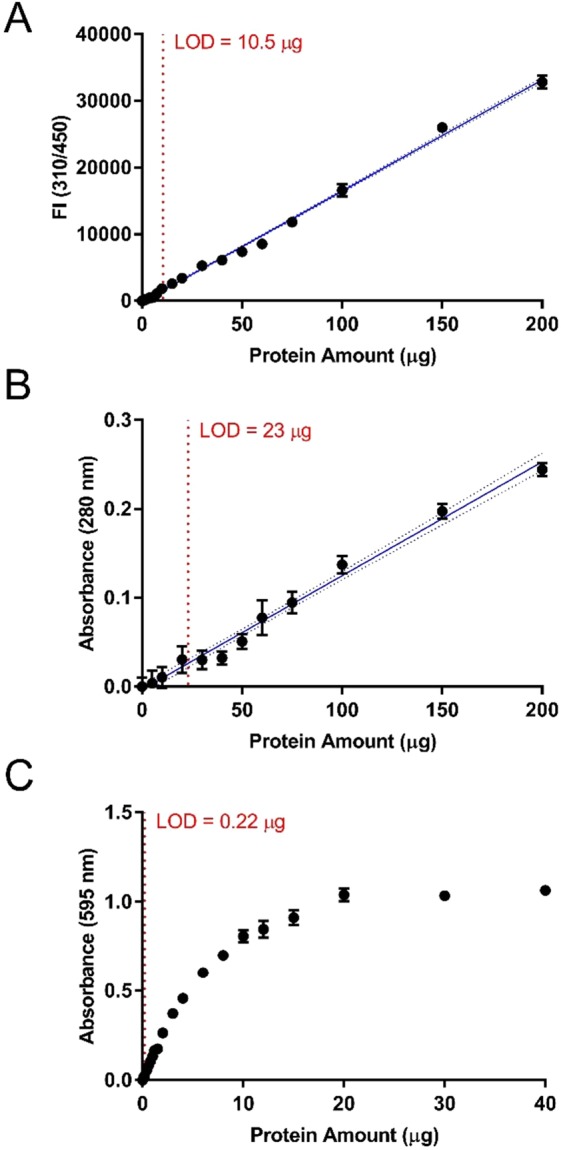
Standard curves for several protein quantification methods. Standard curves of protein amount via (**A**) TCE, (**B**) A280, and (**C**) Bradford assays. (**A**) Fluorescence intensity (FI; emission ʎ = 450 nm; excitation ʎ = 310 nm) of 0–200 µg BSA following 15 minutes of UV-exposure in the presence of 0.5% (v/v) TCE. (**B**) Absorbance measurements (ʎ = 280 nm) of 0–200 µg BSA. (**C**) Absorbance measurements (ʎ = 595 nm) of 0–40 µg BSA in Bradford reagent. Error bars represent propagated standard deviations of blank corrected means. The interpolated standard curve and 95% confidence intervals are represented by solid and dotted blue lines, respectively. The limit of detection (LOD) is depicted by the red dotted lines.

The utility of this UV-dependent TCE-based protein modification was demonstrated through the construction of quantification curves displaying the relationship between protein amount and detectable signal for TCE, Bradford, and A280 assays (Fig. 5). The utility of the Bradford assay was limited as the detectable linear range was found to be restricted to 0.22–3 μg and the signal quickly saturated beyond this range (Fig. 5C). In contrast, the TCE assay had an LOD of 10.5 μg within a linear range up to 200 μg (i.e., the maximum protein amount tested), displaying an LOD 46% lower than the comparable LOD for A280 of 23 μg (Fig. 5A,B). Overall, the TCE-based protein quantification assay was found to be advantageous as a medium to high range protein quantification assay with greater sensitivity in comparison to the standard A280 assay.

### Low-volume TCE assay and SDS-PAGE visualization

**Figure 6:**
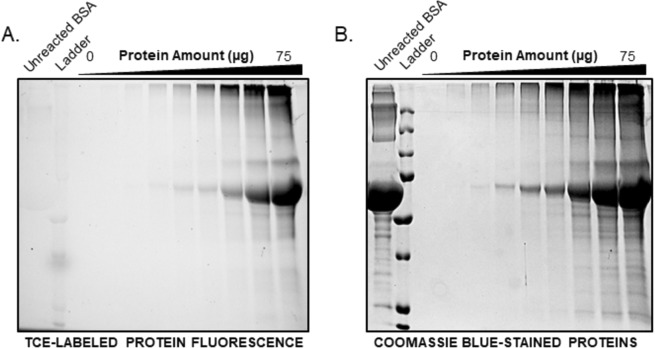
Visualization of TCE-labeled proteins from the low-volume TCE assay following SDS-PAGE. (**A**) Detection of 0–75 µg of BSA loaded from the low-volume TCE assay through UV-light illumination followed by (**B**) Coomassie blue staining of the same gel. The full-sized gels are presented in the supplemental information.

Protein quantification assays which don’t require the use of additional reagents, such as the A280 assay, are an attractive option for small or precious samples as they may be recovered and re-used for downstream applications. Similarly, as the TCE-modification of protein enables visualization in polyacrylamide gels, we aimed to extend the re-use of protein previously used for quantification for SDS-PAGE. To enable practical use of the TCE-modified protein for this application, we developed a low-volume TCE assay with a 20 μL final volume. The low-volume TCE assay maintained greater sensitivity over the A280 assay, displaying an LOD of 8.7 μg after 15 minutes of UV-exposure (Fig. S4). Microplate assay samples were then subsequently subjected to SDS-PAGE. After separation of the TCE-modified protein by SDS-PAGE, the modified protein was successfully resolved and visualized upon UV-illumination in comparison to an unlabeled control BSA sample (Fig. 6A). Coomassie staining of the gel, post SDS-PAGE, served as visual confirmation and control of protein loading (Fig. 6B). Thus, the low-volume TCE assay enables sample use and direct visualization in SDS-PAGE analysis. As previous reports utilize TCE incorporated into the SDS-PAGE gel matrix during gel casting, the use of this visualization method with most commercially available pre-cast gels would be restricted to procedures involving soaking of the gel in TCE solutions post-electrophoresis. In contrast, the methodology outlined in this study involving the pre-electrophoresis labeling and quantification of proteins with TCE, may present an attractive option due to the volume of TCE saved in comparison to soaking of gels in millilitre volumes of solution.

## Conclusion

We have presented a new microplate format protein quantification assay based on the covalent modification of protein with TCE. The method allows researchers to quantify precious samples using an assay more sensitive than the standard A280 method while maintaining the option of sample re-use for SDS-PAGE. Finally, the potential of TCE-labeling pre-electrophoresis enables practical use of this fluorescent visualization method with both homemade and pre-cast polyacrylamide gels, which may further its use as a visualization method for applications such as two-dimensional gel spot analysis or total protein normalization prior to immunoblot transfer.

## Supplementary information

Supplemental data
