# Supplementary material for: Protein quantification and visualization via ultraviolet-dependent labeling with 2,2,2-trichloroethanol
Source: Sci Rep. 2019 Sep 26;9:13923. doi: 10.1038/s41598-019-50385-9 (PMC6763483; doi:10.1038/s41598-019-50385-9)
Supplement: Supplementary file 1 — Supplemental data [file 41598_2019_50385_MOESM1_ESM.docx]

**Supplemental Information**

**Protein quantification and visualization *via* ultraviolet-dependent labeling with 2,2,2-trichloroethanol**

Anand Chopra^1^, William G Willmore^1, 2, 3 *^, & Kyle K Biggar^1, 2, *^

**Affiliations**

^1^Department of Biology, Carleton University, 1125 Colonel By Dr, Ottawa, ON, K1S 5B6, Canada

^2^Institute of Biochemistry, Carleton University, 1125 Colonel By Dr, Ottawa, ON, K1S 5B6, Canada

^3^Department of Chemistry, Carleton University, 1125 Colonel By Dr, Ottawa, ON, K1S 5B6, Canada

**Supplemental Methods**

The limit of detection (LOD) was calculated using Equation 1, where m and σ represent the slope and the error on the lowest point of the curve, respectively.

Equation 1: 𝐿𝑂𝐷=3.3𝜎/𝑚

**Supplemental Figures**


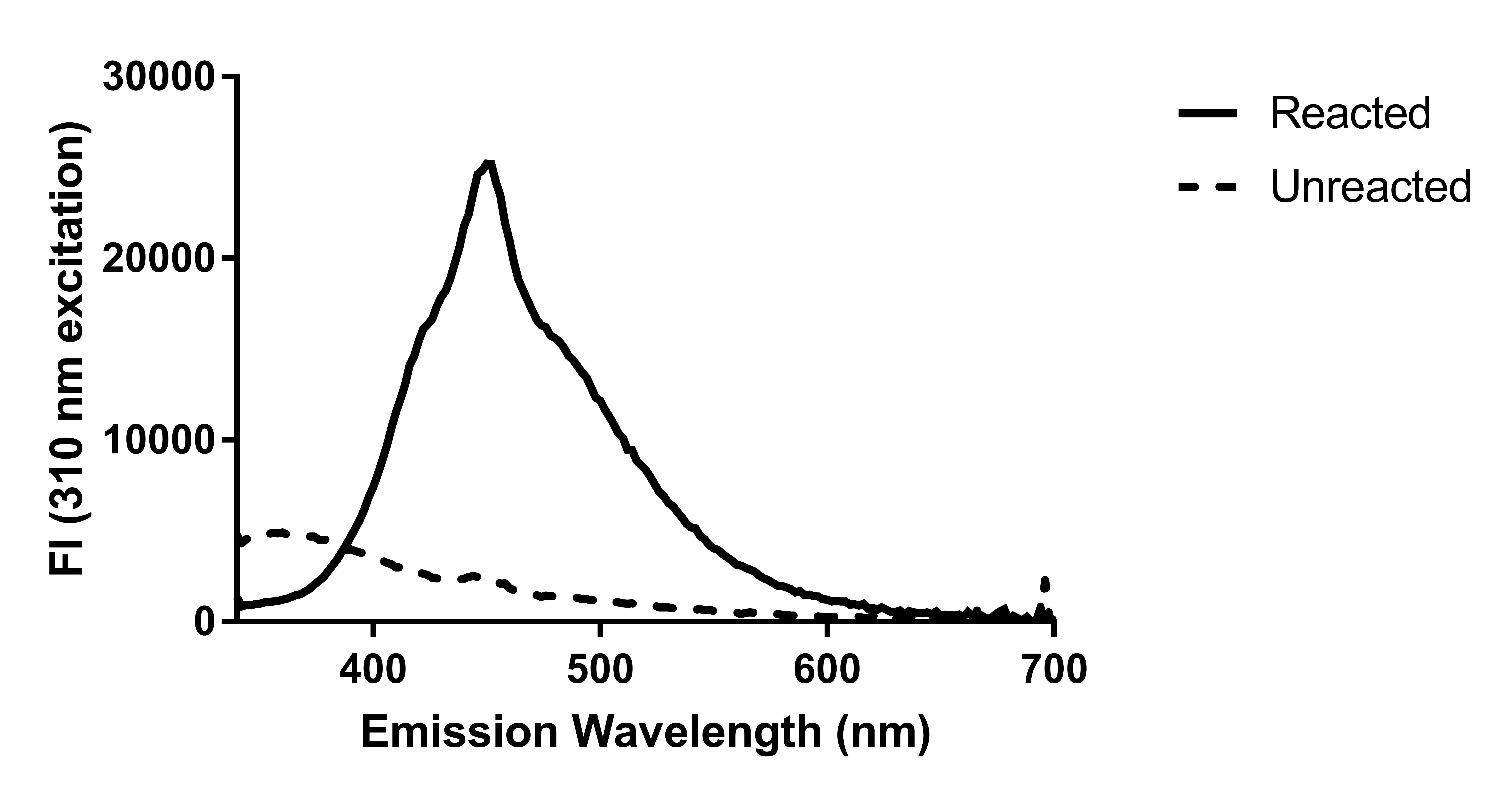


**Figure S1. Emission spectra of unreacted (dashed lines) and TCE-reacted (solid lines) BSA.** Emission spectra (emission ʎ = 340 – 700 nm; excitation ʎ = 310 nm) were collected following 15 minutes of UV-exposure of 1 µg/µL BSA in the presence or absence of 0.5 % (v/v) TCE.


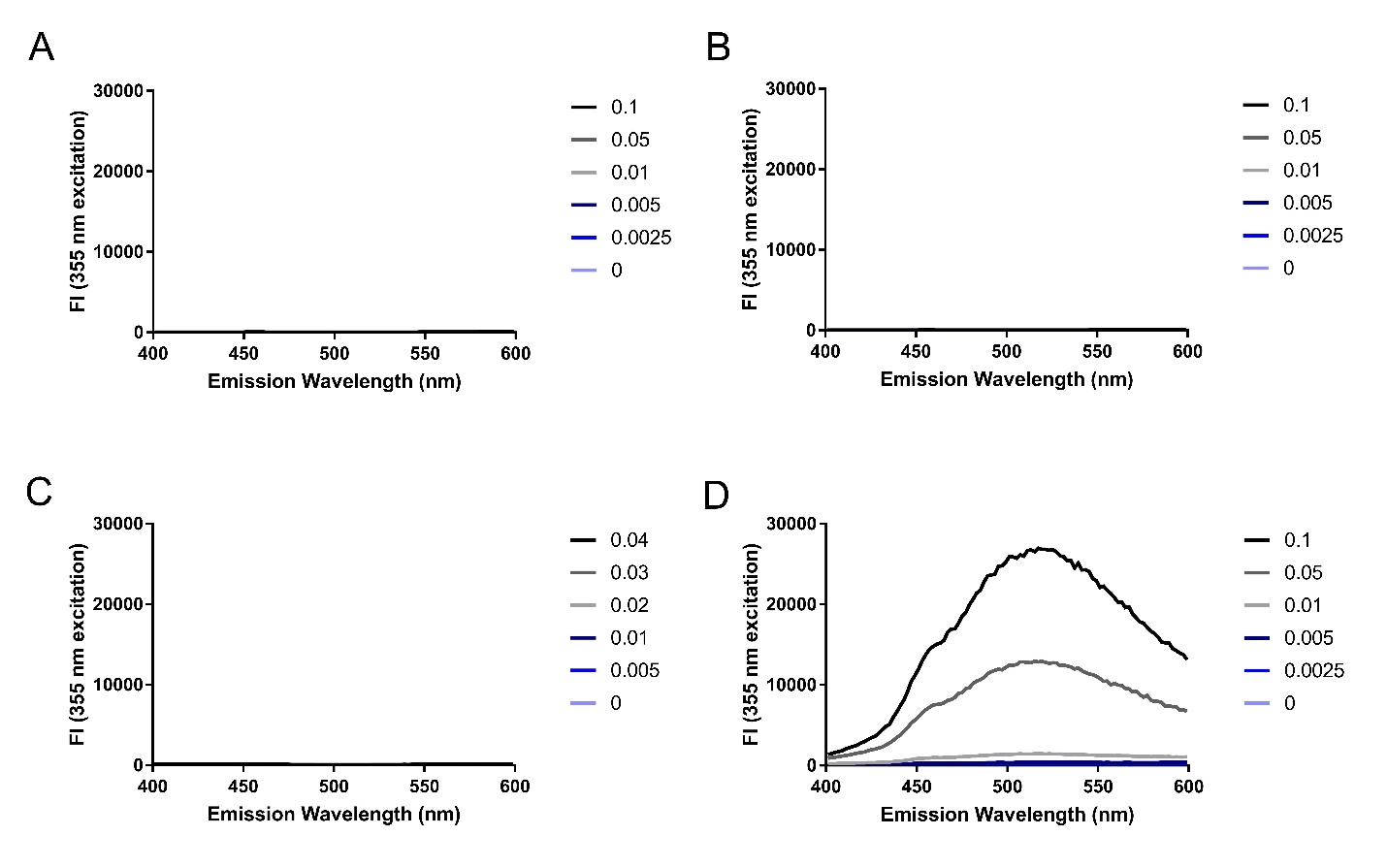


**Figure S2. Emission spectra of individual amino acids following UV-exposure in the presence of TCE.** Information for emission spectra of (a) glycine, (b) phenylalanine, (c) tyrosine, and (d) tryptophan are as described in Figure 3 except for altered fluorescence intensity specifications (emission ʎ = 400 – 600 nm; excitation ʎ = 355 nm).


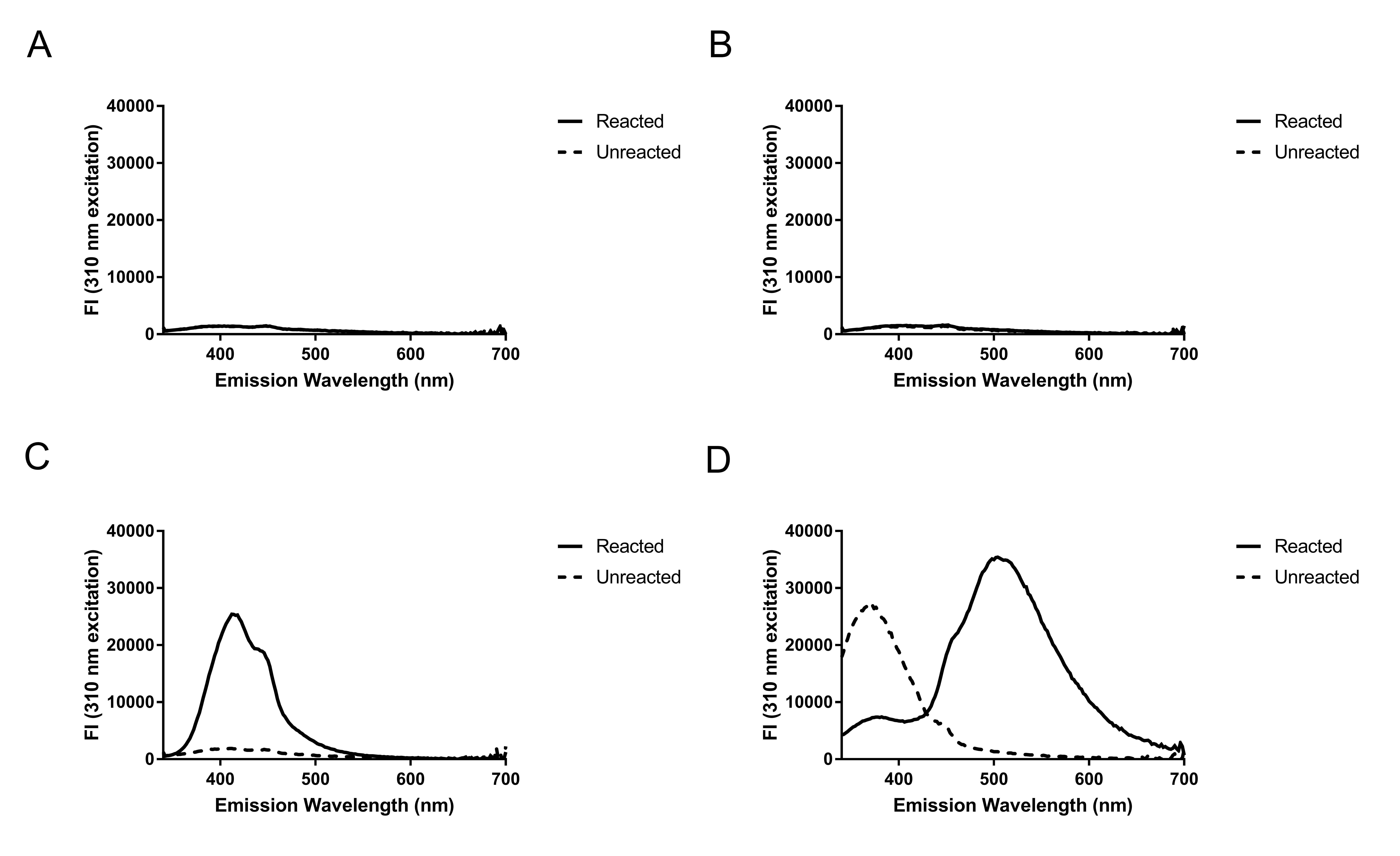


**Figure S3. Emission spectra of unreacted (dashed lines) and TCE-reacted (solid lines) amino acids.** Emission spectra (emission ʎ = 340 – 700 nm; excitation ʎ = 310 nm) of (a) glycine, (b) phenylalanine, (c) tyrosine, and (d) tryptophan were collected following 15 minutes of UV-exposure in the presence or absence of 0.5 % (v/v) TCE. Glycine, phenylalanine, and tryptophan were reacted at final concentration of 0.1 µg/µL whereas tyrosine was at 0.04 µg/µL.


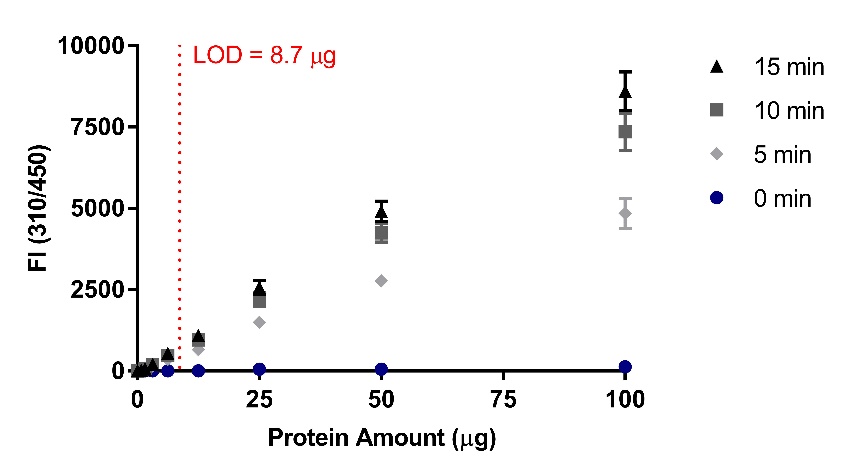


**Figure S4. A low-volume TCE assay.** Fluorescence intensity (FI; emission ʎ = 450 nm; excitation ʎ = 310 nm) of 0 – 100 µg BSA following 0 – 15 minutes of UV-exposure in the presence of 2.5 % (v/v) TCE. The limit of detection (LOD) is depicted by the red dotted line.


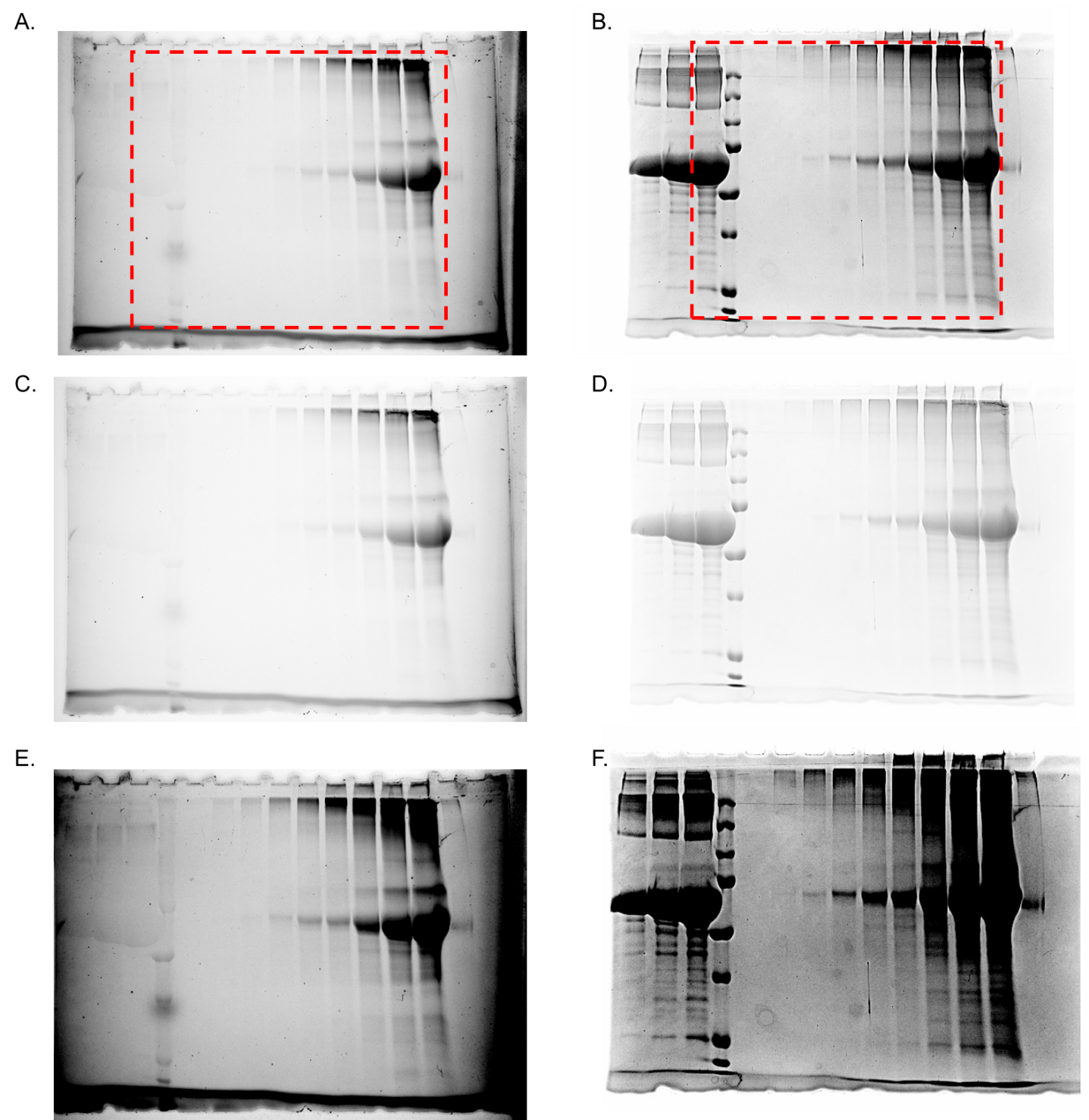


**Figure S5. Full-length gels and multiple contrasts for Figure 6.** Subpanels A and B display the gels as presented in Figure 6, indicating cropped sections by the red-dotted lines. Subpanels C and E are the same image as subpanel A except with different contrasts. Subpanels D and F are the same image as subpanel B except with different contrasts.
